# Poor sleep pattern is associated with metabolic disorder during transition from adolescence to adulthood

**DOI:** 10.3389/fendo.2023.1088135

**Published:** 2023-03-22

**Authors:** Dan Zhang, Yajuan Yang, Shuang Zhai, Yang Qu, Tingting Li, Yang Xie, Shuman Tao, Liwei Zou, Fangbiao Tao, Xiaoyan Wu

**Affiliations:** ^1^ Department of Maternal, Child and Adolescent Health, School of Public Health, Anhui Medical University, Hefei, China; ^2^ MOE Key Laboratory of Population Health Across Life Cycle, Hefei, China; ^3^ NHC Key Laboratory of Study on Abnormal Gametes and Reproductive Tract, Hefei, China; ^4^ Anhui Provincial Key Laboratory of Population Health and Aristogenics, Anhui Medical University, Hefei, China; ^5^ School of Nursing, Anhui Medical University, Hefei, Anhui, China

**Keywords:** sleep pattern, metabolic disorder, adolescence, adulthood, cohort study

## Abstract

**Objective:**

The purpose of this study was to investigate whether sleep pattern is associated with metabolic disorders among young adults.

**Methods:**

We measured sleep patterns using multiple sleep behaviors in an ongoing prospective cohort among college students (n = 1,151). At baseline, 729 college students provided fasting blood samples and human body morphological measurements for quantification of metabolic parameters. Then, 340 participants continued to take metabolic parameters measurements at a 2-year follow-up. Sleep patterns were defined by chronotype, sleep duration, insomnia, snoring, and daytime sleepiness. Metabolic scores were derived for four metabolic parameters including body mass index (BMI), waist circumference (WC), fasting blood sugar (FBG), and insulin. Multivariate linear regression model was applied to analyze the association between sleep pattern types and metabolic parameters and metabolic scores.

**Results:**

In the baseline survey, we found that a total of 41 (4.1%) participants had poor sleep patterns. Then, metabolic scores were significantly higher among college students with poor sleep patterns, compared with those who with healthy sleep patterns at baseline (1.00 ± 0.96 *vs.* 0.78 ± 0.72, *p* < 0.05) and 2-year follow-up (0.34 ± 0.65 *vs.* 1.50 ± 1.64, *p* < 0.05). After covariates were adjusted, poor sleep pattern (*β* = 0.22, 95% CI: 0.06~2.53, *p* = 0.001) was associated with elevated metabolic scores at the 2-year follow-up.

**Conclusions:**

The elevated metabolic burden observed in college students with poor sleep patterns highlights the need to identify and address sleep problems in order to minimize the long-term impact on disease vulnerability.

## 1 Introduction

Metabolic disorders, a global public health issue of great concern (1), are associated with a wide range of chronic conditions including type 2 diabetes mellitus, abdominal obesity, and increased risks of mortality. Over the last two decades, identifying behaviors during early life that may promote metabolic health has come to the forefront of both public health and scientific interest (1). Remarkably, metabolic disorders in young adults can promote metabolic syndrome (MetS) and other chronic non-communicable diseases in later life (2), and more than half of university students have metabolic risk factors (3). Therefore, interventions early in life are essential. The young adult stage is a particularly critical developmental period for the emergence of behaviors that increase the risk for metabolic (4), and this period is an essential window to identify modifiable novel risk factors and to intervene to prevent metabolic disorders later in life. Unfortunately, there are fewer studies on identifying behavioral risk factors for metabolic disorders during this period.

Emerging evidence has implicated that various sleep problems have been identified as modifiable risk factors for metabolic health.[5~9] In young adults, both evening chronotypes (5) and short sleep duration (6) are associated with metabolic abnormalities. There is also emerging literature demonstrating a relationship between excessive daytime sleepiness and MetS (7). In addition, snoring (8) and insomnia (9) are risk factors for metabolic disorders in children and adolescents. These sleep behaviors are intricately linked and may affect in a concerted manner. Sometimes, modifications in one sleep behavior usually lead to compensatory changes in other sleep behaviors (10). However, in most of the previous studies,[5~9] sleep behaviors were assessed individually, without taking into account the complexity and correlations of various sleep behaviors among individuals. Therefore, Li X proposed a comprehensive assessment of sleep patterns using five indicators including excessive daytime sleepiness, snoring, insomnia, short sleep duration, and evening chronotype (10).

To address these key evidence gaps, we measured multiple sleep behaviors and metabolic parameters for 2 years in a prospective follow-up study among young adults. Our cohort study design also would provide novel insights into the role of circadian rhythm in the development of metabolic health. Consequently, we formulated two hypotheses: 1) there is a positive association between poor sleep patterns and metabolic parameters disorder; 2) poorer sleep patterns may predict the risk of metabolic disorders 2 years later.

## 2 Methods

### 2.1 Participants

**Figure 1:**
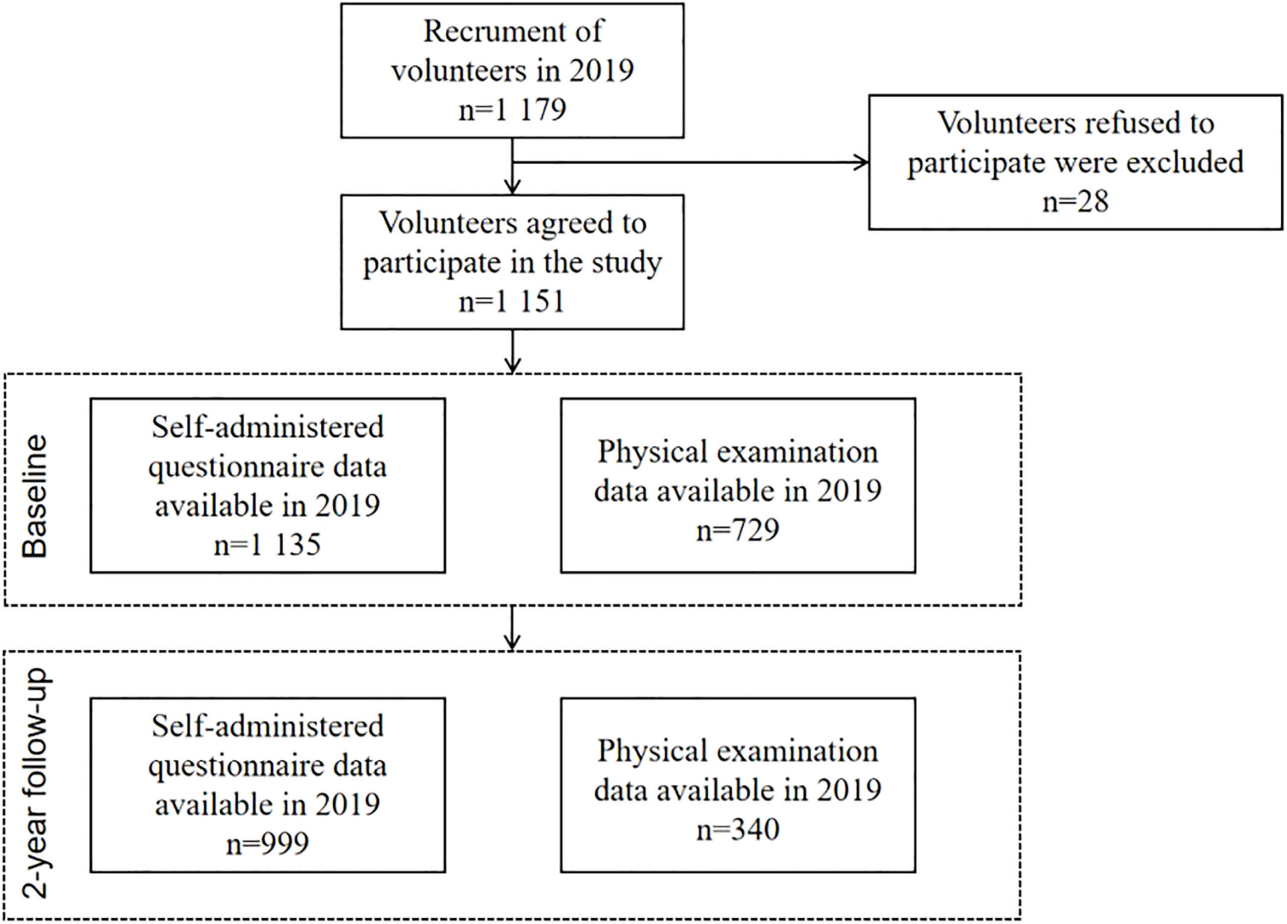
Flowchart of sample populations in our study.

College Student Behavior and Health Cohort Study (11) is a cohort study designed to investigate the longitudinal outcomes of health-related behaviors of college students who were in freshman through junior year between April 2019 and June 2021. The survey was conducted using a cluster of a random sampling of whole groups with the college as the primary sampling unit. First, participants were chosen from a medical university situated in Hefei, Anhui Province, and a comprehensive university located in Shangrao, Jiangxi Province. Among the 1,179 participants at baseline, 1,135 college students (97.6%) completed the self-administered questionnaire, and 729 (61.8%) attended the physical examination. At the 2-year follow-up, 999 participants completed a self-administered questionnaire, and among them, 340 attended the physical examination; see **Figure 1** for details.

The study protocol was approved by the Ethics Committee of Anhui Medical University (No. 20170291). In accordance with the principles of the Declaration of Helsinki, written informed consent was obtained from all participants prior to the completion of the survey. Upon completion of the survey, a free physical examination report was provided to all study participants.

### 2.2 Sleep pattern

At baseline, an electronic questionnaire was used to assess several sleep behaviors in participants, and then an index score of sleep pattern was established, which includes five aspects of sleep: excessive daytime sleepiness, snoring, insomnia, sleep duration, and chronotype (10).

#### 2.2.1 Excessive daytime sleepiness

Excessive daytime sleepiness was assessed using the Pittsburgh Sleep Quality Index (PSQI) (12), which is a component of PSQI and scored on a scale from 0 to 3. A higher score means that excessive daytime sleepiness is more severe.

#### 2.2.2 Snoring

Snoring was assessed by a question from the PSQI (“Do you snore or cough in the middle of the night?: 1) No; 2) Yes”) (12).

#### 2.2.3 Insomnia

Insomnia Severity Index (ISI) was used to assess insomnia symptoms.[132] The ISI is a self-report scale aimed at assessing the severity of insomnia over the past 2 weeks, with total scores ranging from 0 to 28. The higher the score, the more severe the insomnia symptom, with a score of ≥9 defined as insomnia and <9 defined as no insomnia.

#### 2.2.4 Sleep duration

Sleep duration was reported as the number of hours spent sleeping during the day (including naps).

#### 2.2.5 Chronotype

Chronotype was evaluated by this question (“What chronotype do you think you are: 1) definitely a ‘morning’ person; 2) more a ‘morning’ person than ‘evening’ person; 3) more an ‘evening’ person than a ‘morning’ person; 4) definitely an ‘evening’ person.”) from r-Morning and Evening Questionnaire (rMEQ) (13).

Healthiest Sleep pattern was defined as early chronotype (“morningness” or “morningness than eveningness”), normal sleep duration (7–8 h/day), no insomnia, no snoring, and no excessive daytime sleepiness (“never/rarely” or “sometimes”). Each sleep behavior was coded 1 if fitting the healthy criterion and 0 if not (see **Table A1** for details). The healthy sleep score was obtained by summing up the five individual sleep behaviors. A higher score indicated a healthier sleep pattern, with a score of ≥4 defined as healthy, 2~3 defined as intermediate, and ≤1 defined as poor, and analyzed separately as categorical variables.

### 2.3 Metabolic health

The participants of this study were sent to a local Grade III Level A hospital for medical examination and measurement of height and weight using a fully automatic electronic height and weight meter. Body mass index (BMI) was calculated as weight in kilograms divided by the square of height in meters. Waist circumference (WC) measurement is accurate to 0.1 cm. The participants provided 5 ml of fasting venous blood, and fasting blood sugar (FBG) and insulin were measured.

Waist circumference was defined by the high waist circumference screening threshold among children and adolescents aged 7~18 years (WS/T 611-2018) (14). The threshold of BMI was defined using the screening for overweight and obesity among school-age children and adolescents (WS/T 586-2018) (15). The other parameters refer to the demarcation standard of university students in our previous study (11).

Metabolic scores were calculated using four metabolic parameters including BMI, WC, FBG, and insulin, which were mentioned in our previous study (11). Each parameter is defined as a value according to the threshold criteria; parameters above the threshold are defined as a score of 1. The total metabolic score was obtained by the sum of four metabolic parameters, and the range of score was 0~4. The higher the score, the more serious the metabolic disorders were. In addition, several metabolic parameters were transformed as standardized and analyzed separately as continuous variables.

### 2.4 Covariates

Covariates included alcohol consumption, cigarette consumption, mobile phone addiction, and physical activity. In the Young Risk Behavior Surveillance System questionnaire, two questions were modified to measure current cigarette and alcohol consumption (16). Cigarette consumption was assessed by “In the past month, how many days have you smoked?” Alcohol consumption was assessed by “In the past month, how many days have you drunk?” The answers are recoded as “yes” or “no”. The Self-Assessment Questionnaire for Adolescents with Problematic Mobile Phone Use (SQAPMPU) is a standardized questionnaire developed by Tao et al. to assess adolescent PMPU (17). Scores ≥27 are defined as having mobile phone addiction. Physical activity was assessed using International Physical Activity Questionnaire Short Form (IPAQ-SF) (18), with level divided into three categories: low, moderate, and high. Both depression and anxiety were assessed using the depression anxiety stress Scale (DASS-21) (19), with level divided into three categories: low, moderate, and high. DASS-21 is a self-reported, globally popular scale consisting of 21 items (including three subscales of depression, anxiety, and stress, each of which contains seven items). The subscale of depression scores >9 are defined as depression symptom, and the subscale of anxiety scores >7 are defined as anxiety symptom.

### 2.5 Statistical analyses

All statistical tests were two-sided, and significance was set at *p* < 0.05. All data were analyzed in SPSS version 23.0, and the graphs were plotted using GraphPad Prism 8. In the present study, continuous variables of the normal distribution were represented as mean and standard deviation (SD), while categorical variables were summarized using frequencies (n) and percentages (%).

First, one-way analysis of variance tests were used to test the differences among different sleep patterns on several metabolic parameters and metabolic scores. Then, multivariate linear regression model was applied to analyze the association between sleep pattern types and metabolic parameters, and metabolic scores. Last, sensitivity analyses were performed to compare the distribution of sleep patterns between the loss-visit and non-loss-visit groups using chi-square tests, and the association between sleep patterns and metabolic parameters was further tested using multiple linear regression after excluding the non-loss-visit group. Standardized *β* coefficients, with 95% CIs, were reported after adjusting for covariates (including gender, cigarette consumption, alcohol consumption, physical activity, mobile phone addiction, depression, and anxiety). Existing findings showed that poor sleep pattern was characterized by worse physical and mental health compared to healthy sleep pattern (10). Therefore, the healthier group and healthy sleep patterns were recognized as the control group.

## 3 Results

The mean age of the 1,135 participants at baseline was 18.79 years (SD = 1.15), and 703 (61.9%) were female. Over forty percent (42.1%) of participants had healthy sleep patterns, and 41 (4.1%) had poor sleep patterns. The demographic and behavioral characteristics and metabolic parameters of participants at baseline and 2-year follow-up are shown in **Table 1**. WC, BMI, insulin, and FBG were significantly higher at the 2-year follow-up compared to the baseline level (**Table 1**). The mean metabolic score was 0.97 (SD = 0.77) at baseline and 0.49 (SD = 0.80) at the 2-year follow-up.

**Table 1:** Descriptive statistics of demographic, behavior characteristics, and metabolic parameters.

Characteristics	Baseline		2-year follow-up	
	*N*	Mean ± SD (%)	*N*	Mean ± SD (%)
Demographic and behavioral characteristics				
Age (years)	1,135	18.79 ± 1.15	999	20.75 ± 0.90
Gender, female	703	61.9	622	62.3
Cigarette consumption	101	8.9	96	9.6
Alcohol consumption	261	23.0	229	22.9
Low physical activity	166	14.6	178	17.8
Mobile phone addiction	279	24.6	297	29.7
Depression	244	21.5	236	23.6
Anxiety	329	29.0	224	22.4
Poor sleep pattern	41	4.1	–	
Metabolic				
WC (cm)	729	71.06 ± 7.80	340	73.77 ± 9.64
BMI (kg/m^2^)	729	20.76 ± 2.88	340	21.20 ± 3.17
Insulin (uIU/ml)	729	5.46 ± 4.53	340	11.31 ± 8.75
FBG (mmol/L)	729	4.55 ± 0.41	340	4.61 ± 0.39
Metabolic score	729	0.97 ± 0.77	340	0.49 ± 0.80

SD, standard deviation; WC, waist circumference; BMI, body mass index; FBG, fasting blood sugar; -, Not investigated.

At baseline, compared to those with healthy sleep patterns, participants with poor sleep patterns had higher levels of WC (75.33 *vs.* 70.22 cm, *p* < 0.05), while participants with intermediate sleep patterns had a higher level of BMI (1.63 mg/L), insulin (57.83 *vs.* 45.71 uIU/ml, *p* < 0.05) and metabolic scores (1.06 *vs.* 0.78, *p* < 0.05). No elevated levels of FBG were found in different sleep pattern groups (**Table 2**).

**Table 2:** Metabolic parameters according to sleep pattern.

Characteristics	Healthy	Intermediate	Poor	*p*-Value	*F* value
Baseline					
WC (cm)	70.22 ± 6.67	71.46 ± 8.41	75.33 ± 10.71*	0.018	4.050
BMI (kg/m^2^)	20.32 ± 2.32	20.99 ± 3.02*	21.67 ± 3.15	0.006	5.231
Insulin (uIU/ml)	45.71 ± 39.96	57.83 ± 49.28*	64.17 ± 54.73	0.004	5.683
FBG (mmol/L)	4.56 ± 0.42	4.56 ± 0.42	4.50 ± 0.38	0.863	0.124
Metabolic scores	0.78 ± 0.72	1.06 ± 0.80*	1.00 ± 0.96	<0.001	9.413
2-year follow-up					
WC (cm)	72.44 ± 9.64	73.26 ± 10.21	78.83 ± 15.89	0.312	1.171
BMI (kg/m^2^)	20.87 ± 2.73	21.33 ± 3.24	19.40 ± 8.74	0.254	1.377
Insulin (uIU/ml)	10.75 ± 9.20	12.79 ± 9.76	19.74 ± 12.66*	0.044	3.161
FBG (mmol/L)	4.62 ± 0.42	4.68 ± 0.36	4.61 ± 0.59	0.531	0.635
Metabolic scores	0.34 ± 0.65	0.61 ± 0.85*	1.50 ± 1.64*	0.001	7.529

SD, standard deviation; WC, waist circumference; BMI, body mass index; FBG, fasting blood sugar.

^*^ Dunnett’s test with the “healthy sleep pattern” group as the reference group showed statistically significant results.

At the 2-year follow-up, compared to healthy sleep pattern, those participants with poor sleep patterns had higher levels of insulin (57.83 *vs.* 45.71 uIU/ml, *p* < 0.05) and metabolic scores (1.50 *vs.* 0.34, *p* < 0.05), while participants with intermediate sleep pattern had a higher level of metabolic scores (0.61). No elevated levels of other metabolic parameters were found in different sleep pattern groups (**Table 2**).

**Figure 2:**
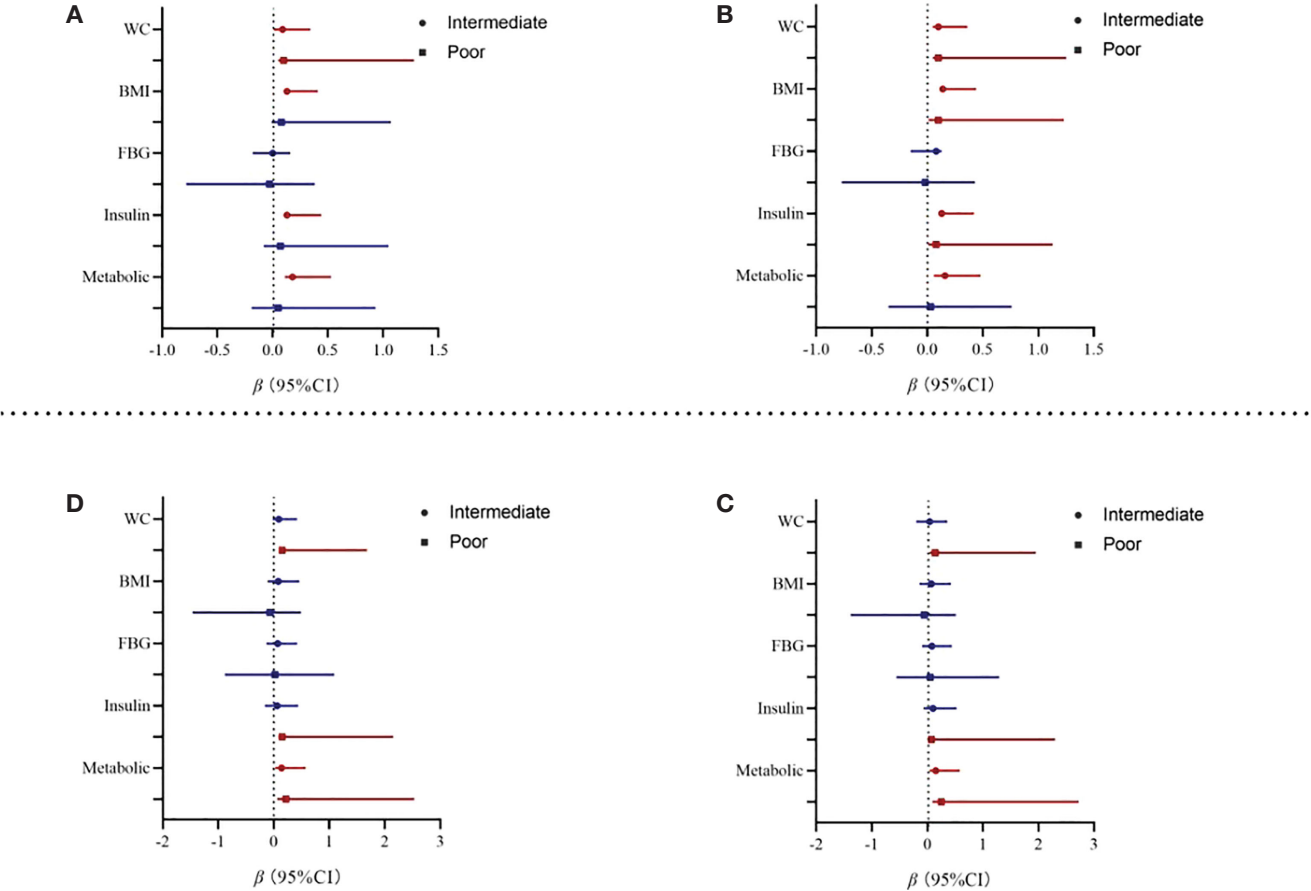
Association from multivariate linear regression model between sleep and metabolic parameters. Note: WC, waist circumference; BMI, body mass index; FBG, fasting blood sugar. Model 2 adjusted for baseline gender, cigarette consumption, alcohol consumption, physical activity, mobile phone addiction, depression, and anxiety. **(A)** Baseline Model 1. **(B)** Baseline Model 2. **(C)** Two-year follow-up Model 1. **(D)** Two-year follow-up Model 2.

At baseline, after baseline gender, cigarette consumption, alcohol consumption, physical activity, mobile phone addiction, depression, and anxiety were adjusted, poor sleep pattern was positively associated with elevated WC (*β* = 0.10; 95% CI: 0.05~1.25), BMI (*β* = 0.10; 95% CI: 0.01~1.23), and insulin (*β* = 0.08; 95% CI: 0.01~1.13). The positive association between intermediate sleep patterns with elevated levels of WC, BMI, and insulin was kept statistically significant after adjustment for covariates (see **Figure 2**; **Table A2**). In addition, intermediate sleep pattern was positively associated with elevated metabolic scores (*β* = 0.16; 95% CI: 0.06~0.48).

At the 2-year follow-up, after we adjusted for baseline covariates, participants with poor sleep patterns were associated with increased 0.22 units in metabolic scores (95% CI: 0.06~2.53). In addition, poor sleep pattern was also positively associated with elevated WC (*β* = 0.13; 95% CI: 0.15~1.68) and insulin (*β* = 0.15; 95% CI: 0.08~2.15). However, the association between intermediate sleep patterns with elevated levels of WC and insulin disappeared after adjustment for covariates, see in **Table 3**. Results from the sensitivity analyses are shown in **Table A3**; we first compared the baseline sleep patterns of the loss-visit and non-loss-visit groups, and the results were not statistically significant. Then, we excluded the loss-visit group and analyzed the association between sleep patterns and metabolic indicators and metabolic scores at the baseline survey, and the majority of the results remained consistent as above.

**Table 3:** Association from multivariate linear regression model between sleep pattern and metabolic parameters at 2-year follow-up.

	Characteristics	Model 1		Model 2	
		*β* (95% CI)	*p*	*β* (95% CI)	*p*
WC	Healthy	Ref.		Ref.	
	Intermediate	0.04 (−0.20 to 0.36)	0.575	0.09 (−0.02 to 0.42)	0.073
	Poor	0.14 (0.04 to 1.95)	0.042	0.13 (0.15 to 1.68)	0.019
BMI	Healthy	Ref.		Ref.	
	Intermediate	0.07 (−0.14 to 0.42)	0.328	0.08 (−0.11 to 0.46)	0.221
	Poor	−0.06 (−1.38 to 0.51)	0.362	−0.07 (−1.46 to 0.49)	0.330
FBG	Healthy	Ref.		Ref.	
	Intermediate	0.08 (−0.10 to 0.44)	0.222	0.07 (−0.13 to 0.42)	0.306
	Poor	0.05 (−0.56 to 1.29)	0.433	0.02 (−0.88 to 1.09)	0.777
Insulin	Healthy	Ref.		Ref.	
	Intermediate	0.10 (−0.07 to 0.52)	0.142	0.06 (−0.16 to 0.44)	0.350
	Poor	0.17 (0.03 to 2.30)	0.011	0.15 (0.08 to 2.15)	0.034
Metabolic score	Healthy	Ref.		Ref.	
	Intermediate	0.15 (0.04 to 0.58)	0.024	0.14 (0.03 to 0.57)	0.028
	Poor	0.25 (0.09 to 2.72)	<0.001	0.22 (0.06 to 2.53)	0.001

Model 2 adjusted for baseline gender, cigarette consumption, alcohol consumption, physical activity, mobile phone addiction, depression, and anxiety.

WC, waist circumference; BMI, body mass index; FBG, fasting blood sugar.

## 4 Discussion

In this prospective cohort study of 1,151 Chinese young adults, poor sleep patterns were significantly associated with higher metabolic scores, even after adjustment for known metabolic risk factors. Specifically, those participants with baseline poor sleep patterns were associated with increased 0.22 units in metabolic scores at 2-year follow-up. Moreover, poor sleep pattern was associated with disrupted insulin homeostasis (elevated FBG) and obesity (elevated WC and BMI). These findings suggest that improved sleep patterns can help promote cardiovascular metabolic health.

In the present study, 4.1% of the study subjects showed poor sleep patterns, and these subjects had a metabolic score of 1.50 ± 1.64 at 2 years. We found that poor sleep pattern was associated with disrupted insulin homeostasis (elevated FBG) and obesity (elevated WC and BMI); this result is consistent with evidence from previous studies. Skjakodegard HF et al. reported that later sleep timing was related to obesogenic behaviors in children and may represent an obesity risk factor (20). Moreover, Van Dijk D et al. also assessed that sleep plays an important role in insulin resistance among middle-aged people and may provide a link to the development of type 2 diabetes (21). Our study highlights the impact of freshman sleep behavior on metabolic health after 2 years. The present study also showed a higher metabolic risk among college students, which is also in line with previous studies (3). The freshman phase of college is a critical period of behavioral development during which undesirable behaviors that affect metabolic parameters may develop, thereby adversely affecting future metabolic health. Currently, there has been a growing body of studies suggesting that sleep behaviors are associated with metabolic health.[5~9] Our findings are broadly consistent with previous studies but extended to combine various sleep behaviors. Indeed, sleep patterns combining several sleep behaviors are more reflective of an individual’s true sleep status, as a variety of sleep behaviors are intricately linked (10). Currently, these sleep patterns are widely used in middle-aged and older adults, while no studies on young adults are available. However, several studies have shown that the sleep indicators associated with these sleep patterns in young adults are not promising (5–9). In addition, a continuous metabolic score was used to estimate metabolic health in a comprehensive manner in the present study through a paradigm shift from the “treatment of risk factors in isolation” to “comprehensive metabolic risk management”, which may better reflect the metabolic health of the individual. Compared with traditional single clinical indicators, the composite indicator of metabolic scores could help in more accurate predictions of chronic condition risk in young adults.

Several potential mechanisms could explain the observed associations between poor sleep patterns and the increased risk of metabolic health in the present study (22, 23). First, a compelling body of evidence demonstrates that sleep is a physiologic state of decreased global metabolism, which likely serves a reparative role, and growth hormone (GH) is secreted in the first few hours of a usual sleep period, which may serve to spare the catabolism of protein and glucose stores. Second, circadian rhythms, dependent on sleep, also affect hormone profiles and metabolism. Mild diurnal fluctuations in some hormones including glucose also occurred. Then, circadian misalignment caused significant metabolic disorders. Lastly, sleep loss also affects appetite and food intake, thereby promoting obesity.

This study has limitations. First, due to oversight at the time of the survey, there were a number of confounding factors, including dietary factors, that were not included in the analysis and could be added to further refine the study findings in the future. Second, the study participants were all college students, which have their own group characteristics, so the study may have selection bias, and the results to the general population extrapolation may be limited. Third, sleep patterns for this study were collected at baseline, and sleep pattern profiles are subject to change over 2 years, which may result in less reliable study findings. At last, at the time of follow-up, most participants were missing metabolic index data due to epidemic prevention and control; however, we performed a sensitivity analysis for both surveys, thus ensuring the reliability of the findings.

Nonetheless, the current study has several strengths. First, this study is the first prospective study examining associations between sleep patterns and metabolic health in young adults. Second, the sleep patterns used by this study were combined with various sleep behaviors and provided a better frame of reference for sleep. Last, we used a combination of metabolic index data to truly reflect the metabolic health status of individuals.

## 5 Conclusions

The current study was conducted in a cohort of young adults and combined multiple sleep behaviors to assess sleep patterns, indicating that poor sleep pattern was associated with higher metabolic risk even after accounting for known risk factors. Further research is needed to confirm our findings and to elucidate potential mechanisms.

## Data availability statement

The original contributions presented in the study are included in the article/**Supplementary Material**. Further inquiries can be directed to the corresponding author.

## Author contributions

XW and FT conceived and designed the experiments. YY, ST and LZ performed the experiments. DZ analyzed the data. LZ, YQ, SZ, TL, and YX contributed reagents, materials, and analysis tools. DZ wrote the paper. DZ contributed to the study design. All authors contributed to the article and approved the submitted version.
