# Supplementary material for: Poor sleep pattern is associated with metabolic disorder during transition from adolescence to adulthood
Source: Front Endocrinol (Lausanne). 2023 Mar 22;14:1088135. doi: 10.3389/fendo.2023.1088135 (PMC10073678; doi:10.3389/fendo.2023.1088135)
Supplement: Supplementary file 1 [file DataSheet_1.docx]

**Poor sleep pattern is associated with metabolic disorder in young adults**

**Table A.1.** Development of healthy sleep pattern scores for Chinese college students

| **Risk Indexes** | **Score** |
| --- | --- |
|  |  |
| **Chronotype** |  |
| Morningness | 1 |
| Morningness than eveningness | 1 |
| Eveningness than morningness | 0 |
| Eveningness | 0 |
| **Sleep duration** |  |
| Normal (7–8 h) | 1 |
| Short (<7 h) | 0 |
| Long (>8 h) | 0 |
| **Insomnia** |  |
| No | 1 |
| Yes | 0 |
| **Snoring** |  |
| No | 1 |
| Yes | 0 |
| **Excessive daytime sleepiness** |  |
| Never/rarely(0) | 1 |
| Sometimes(1) | 1 |
| Often(2) | 0 |
| All of the time(3) | 0 |
| **Healthy sleep pattern** | 0~5 |

**Table A.2.** Association from multivariate linear regression model between sleep pattern and metabolic parameters at baseline.

|  | Characteristics | Model 1 | |  | Model 2 | |
| --- | --- | --- | --- | --- | --- | --- |
|  |  | *β* (95%*CI*) | *P* |  | *β* (95%*CI*) | *P* |
| WC | Healthy | Ref. |  |  | Ref. |  |
|  | Intermediate | 0.09 (0.01~0.34) | 0.033 |  | 0.10 (0.05~0.36) | 0.011 |
|  | Poor | 0.10 (0.05~1.28) | 0.013 |  | 0.10 (0.05~1.25) | 0.014 |
| BMI | Healthy | Ref. |  |  | Ref. |  |
|  | Intermediate | 0.13 (0.10~0.41) | 0.001 |  | 0.14 (0.12~0.44) | 0.001 |
|  | Poor | 0.08 (-0.01~1.07) | 0.054 |  | 0.10 (0.01~1.23) | 0.002 |
| FBG | Healthy | Ref. |  |  | Ref. |  |
|  | Intermediate | 0.00 (-0.18~0.16) | 0.941 |  | 0.08 (-0.15~0.13) | 0.851 |
|  | Poor | -0.03 (-0.78~0.38) | 0.493 |  | -0.02 (-0.77~0.43) | 0.584 |
| Insulin | Healthy | Ref. |  |  | Ref. |  |
|  | Intermediate | 0.13 (0.11~0.44) | 0.001 |  | 0.13 (0.10~0.42) | 0.001 |
|  | Poor | 0.07 (-0.08~1.05) | 0.093 |  | 0.08 (0.01~1.13) | 0.045 |
| Metabolic scores | Healthy | Ref. |  |  | Ref. |  |
|  | Intermediate | 0.18 (0.11~0.53) | <0.001 |  | 0.16 (0.06~0.48) | <0.001 |
|  | Poor | 0.05 (-0.19~0.93) | 0.190 |  | 0.03 (-0.35~0.76) | 0.464 |

Note: *CI*, confidence interval; WC, waist circumference; BMI, Body mass index; FBG, fasting blood sugar; Model 2 adjusted for baseline gender, cigarette consumption, alcohol consumption, physical activity, mobile phone addition, depression and anxiety.

**Table A.3.** Sensitivity analysis about the association between sleep patterns and metabolic parameters among non-loss-visit group

|  | Characteristics | Model 1 | |  | Model 2 | |
| --- | --- | --- | --- | --- | --- | --- |
|  |  | *β* (95%*CI*) | *P* |  | *β* (95%*CI*) | *P* |
| WC | Healthy | Ref. |  |  | Ref. |  |
|  | Intermediate | -0.00 (-0.29~0.28) | 0.979 |  | 0.04 (-0.19~0.34) | 0.580 |
|  | Poor | 0.17 (0.28~2.20) | 0.011 |  | 0.17 (0.28~2.19) | 0.011 |
| BMI | Healthy | Ref. |  |  | Ref. |  |
|  | Intermediate | 0.09 (-0.09~0.42) | 0.196 |  | 0.10 (-0.08~0.45) | 0.162 |
|  | Poor | 0.18 (0.31~2.02) | 0.008 |  | 0.17 (0.21~2.05) | 0.017 |
| FBG | Healthy | Ref. |  |  | Ref. |  |
|  | Intermediate | 0.01 (-0.26~0.29) | 0.923 |  | 0.02 (-0.24~0.33) | 0.759 |
|  | Poor | -0.04 (-1.23~0.65) | 0.540 |  | -0.04 (-1.30~0.71) | 0.561 |
| Insulin | Healthy | Ref. |  |  | Ref. |  |
|  | Intermediate | 0.18 (0.10~0.55) | 0.006 |  | 0.17 (0.09~0.52) | 0.007 |
|  | Poor | 0.21 (0.51~2.07) | 0.001 |  | 0.20 (0.41~1.96) | 0.003 |
| Metabolic | Healthy | Ref. |  |  | Ref. |  |
|  | Intermediate | 0.21 (0.14~0.57) | 0.002 |  | 0.19 (0.12~0.54) | 0.002 |
|  | Poor | 0.16 (0.17~1.63) | 0.016 |  | 0.14 (0.03~1.51) | 0.043 |

Note: *CI*, confidence interval; WC, waist circumference; BMI, Body mass index; FBG, fasting blood sugar; Model 2 adjusted for baseline gender, cigarette consumption, alcohol consumption, physical activity, mobile phone addition, depression and anxiety.
